# Made Useful

**Published:** 1883-09

**Authors:** 


### MADE USEFUL.

There is one man in this city who is willing to be managed by his wife. He knows that she is efficient, and that he is deficient; he therefore relies on her to support the family and to find him employment. They live happily together, as the following narrative of her experience, given in the Boston *Globe*, shows:

I am a milliner, and I have made between $1,500 and $2,500 a year in my business for some time past.

I married four years ago. My husband is kind and good-looking, but he never learned any trade, had no profession, and could not average $500 a year.

I loved him, however, but I saw that it would not do to depend upon him, so I kept on with my business.

After a time I think he became a little lazy, and as we were both away during the day, we could not keep house, and were tired of boarding.

Finally, I proposed that he should keep house and I would run the business and find the money. We have now lived very happily in this way for two years.

My husband gets up and builds the fire, gets breakfast, and I leave at 7.45 for my place of business. He does the washing and ironing, the cleaning, and I do not know of any woman who can do such work any better. He is as neat as wax, and can cook equal to any one in town.

I may be an isolated case, but I think the time has now came when women who have husbands to support should make them do the housework; otherwise they are luxuries we must do without.

SHELLAC is the best cement for jet articles. Smoking the joint makes it black to match.

